# Paradigm shifts: The impact of clinical research on understanding persons with dementia and their care

**DOI:** 10.1093/geroni/igaf122.1251

**Published:** 2025-12-31

**Authors:** Jiska Cohen-Mansfield

**Affiliations:** Tel Aviv University, Tel Aviv, Tel Aviv, Israel

## Abstract

The conceptualization of dementia has traditionally been bleak, assuming that little can be done for individuals with dementia, that their behaviors and life experiences are dictated by the disease and that psychoactive medications must be used to control them – at least until effective medication emerge to prevent, alleviate, or cure the disease. However, the last decades of clinical research have revealed a markedly different perspective – one in which past paradigms give way to new possibilities. This lecture will highlight key paradigm shifts in understanding dementia and potential avenues for improved care, including insights into the etiology of behavioral challenges, the preservation of self-identity, improved methods for detecting pain, and strategies for enhancing well-being. By integrating these advances into practice, we can move beyond limiting assumptions and advance toward a more holistic approach that offers opportunities to enhance the life experiences of people living with dementia, and concomitantly, their caregivers.

